# Free school meals, diet quality and food insecurity in secondary school students: protocol for a multiple-methods study – the CANTEEN study

**DOI:** 10.1136/bmjopen-2025-101428

**Published:** 2025-10-20

**Authors:** Emma Alving-Jessep, Miranda Pallan, Ellie Ansell, Lesley Hamill, Cara McConnell, Desiree McIlwee, Michelle C McKinley, Sarah E Moore, Marie Murphy, Charlotte Neville, Christina O’Neill, Estera Sevel, Peymane Adab, Maria Bryant, Stephanie Chambers, Christopher R Cardwell, Hannah Ensaff, Charlotte Evans, Stephen Reid, Angus Holford, Jennifer Bradley, Suzanne Spence, Jayne Woodside

**Affiliations:** 1 Department of Applied Health Sciences, Murray Learning Centre, University of Birmingham, Birmingham, UK; 2 Centre for Public Health, School of Medicine, Dentistry and Biomedical Sciences, Queen's University Belfast, Belfast, UK; 3 Hull York Medical School, University of York, York, UK; 4 Health Sciences, University of York, York, UK; 5 School of Social and Political Sciences, University of Glasgow, Glasgow, UK; 6 Nutritional Sciences and Epidemiology, School of Food Science and Nutrition, University of Leeds, Leeds, UK; 7 Purple Nutrition, Chesterfield, UK; 8 Larne High School, Larne, UK; 9 Institute for Social and Economic Research, University of Essex, Colchester, UK; 10 Human Nutrition and Exercise Research Centre, Newcastle University, Newcastle upon Tyne, UK

**Keywords:** Food Insecurity, Schools, NUTRITION & DIETETICS

## Abstract

**Abstract:**

**Introduction:**

Food insecurity is increasing in the UK, impacting choice and diet quality. The current means-tested free school meals (FSM) policy was put in place to address dietary inequalities and food insecurity in school children. In secondary schools, approximately 20% of students who are eligible and registered do not take their FSM. Working across a range of schools that have variable levels of FSM uptake, this study aims to evaluate the effectiveness and cost-effectiveness of the current means-tested FSM policy in UK secondary schools on diet and food insecurity outcomes, understand what factors are associated with uptake and test the potential impact of any proposed policy change.

**Methods and analysis:**

Secondary schools (n=32) in both Northern Ireland and the Midlands region of the UK are being recruited into the study. Data will be collected from school staff, governors, students and parents via questionnaires, as well as observational data of school eating environments. Qualitative data will be collected in selected case study schools (n=6–8). Multilevel modelling will be undertaken to evaluate the association between FSM uptake and fruit and vegetable intake, overall diet quality and food insecurity in all students. Economic evaluation will be conducted using a cost–utility approach. The effect of policy change will be modelled and school factors associated with FSM uptake explored using multiple methods.

**Ethics and dissemination:**

Ethical approval has been obtained from Queen’s University Belfast Faculty of Medicine, Health and Life Sciences Research Ethical Committee (MHLS 23_55). Findings will be disseminated to key national and local agencies, to schools through reports and presentations, and to the public through media and open access publications.

**STRENGTHS AND LIMITATIONS OF THIS STUDY**

- The study design includes both quantitative and qualitative research to provide a thorough and in-depth analysis understanding of the effectiveness of free school meals (FSM) policy.
- Economic evaluation will determine the cost-effectiveness of the current means-tested FSM policy.
- Factors that are associated with FSM uptake will also be explored using both quantitative and qualitative methods and gathering data from a wide range of stakeholders.
- Dietary data, gathered from secondary school pupils, are self-reported.
- The current nature of the policy precludes the use of randomised controlled trial methodology, and therefore, the design is observational and cross-sectional in nature.

## Introduction

 The number of households facing food insecurity, a measure of material poverty that encapsulates the experience of not having enough food to eat, is increasing. Approximately 19% of UK children aged under 15 years live in moderately food insecure households.^1^ Recent evidence has shown that since the 2019–2020 global COVID-19 pandemic, there has been a rise of approximately 1 million children in the UK facing food insecurity or worse.^2^ Additionally, the current ‘cost of living crisis’ is placing pressures on UK households, particularly those with low incomes.^3^,^5^

Diet quality is directly related to food insecurity, with those at all levels of severity experiencing lower overall diet quality than the general population,^26^,^8^ which is specifically associated with adverse physical and mental well-being in both adults and children.^9^,^11^ In UK adolescents, diet quality is commonly low, with poor dietary choices leading to high intakes of saturated fats and sugars, and low intakes of fruits, vegetables and fibre, all falling outside the recommended levels.^11 12^ This is of concern as dietary habits formed during adolescence are likely to continue into adulthood and lead to increased risk of obesity and future cardiometabolic disease.^13 14^

To combat food insecurity and reduce the disparity in diet quality in children of low-income households, the UK government implemented a means-tested policy to provide free school meals (FSM) to those who are eligible,^15^ although there is regional variation in the eligibility criteria^16 17^ and a move towards universal provision in younger age groups and in certain regions.^18 19^ Following the pandemic, there has been a steady increase in the number of children eligible for FSM. For example, since 2022, there has been an increase of 75 000 children aged 5–16 years old eligible for FSM in England. A total of 2.1 million children (or 24.6%) were, therefore, eligible for FSM in January 2024.^20^

Being eligible and registered does not necessarily equate to uptake of FSM. Many of those who are eligible and registered do not take up their FSM; in the secondary school setting, approximately 20% of those registered do not take up their FSM.^21^,^23^ The reasons for this are complex; a number of factors have been demonstrated to influence uptake of FSM, including lack of clarity about eligibility, school proactivity around FSM and fluctuations in family circumstances.^24 25^ Although previously thought of as an influencing factor, stigma towards FSM has not consistently been reported as a factor in FSM take-up, with some of the stigma being mitigated by school efforts to provide anonymity to FSM children through cashless payment systems.^25^

Even though the FSM policy is one of the government’s key policies targeting dietary inequalities, almost no evaluation of its impact on diet, food insecurity, health or other outcomes has been conducted. Evidence relating to FSMs and diet outcomes in the UK comes mostly from younger children (4–7 years), where limited evidence suggests that universal FSM programmes can reduce obesity and improve dietary choices.^26^,^28^ A global systematic review^29^ of universal FSM programmes demonstrated positive effects on meal participation, diet quality and academic performance, with some limited evidence of a positive impact on food security. There is almost no evidence on the impact of the current UK means-tested FSM policy on dietary intake and food insecurity in secondary schools. A single cross-sectional study collecting data from n=2660 students aged 11–18 years in two schools in Yorkshire, undertaken more than a decade ago, found that those taking FSMs chose the dish of the day, which tends to be more nutritious, more often than non-FSM students.^30^

Furthermore, there is a paucity of evidence on the economic impact of the current UK FSM policy, with no studies evaluating policy cost-effectiveness.^31^ Food insecurity is being exacerbated by the current cost of living crisis,^32 33^ making it imperative that one of the main government policies to tackle food insecurity, means-tested FSM, is fit for purpose. There has been a call to expand FSM to more or all children at school,^34^ due to the food insecurity experienced in children of households with low income who fall outside of the current eligibility criteria, and it is important to understand the potential impact of this and to have robust data on which policy change could be modelled.

The aim of the CANTEEN study is to evaluate the effectiveness and cost-effectiveness of the current means-tested FSM policy in UK secondary schools on diet and food insecurity outcomes, understand what factors are associated with uptake, and model the potential impact of the proposed policy change.

## Methods and analysis

This is an observational, cross-sectional, multiple-methods study design, with outcomes collected at school, student and parent levels and accompanying economic evaluation. As the FSM policy already exists in the secondary school setting, traditional approaches (eg, a cluster randomised controlled trial) are not possible. The variable levels of FSM uptake will, therefore, be used to explore, both at school and student levels, the association between FSM uptake and dietary and food insecurity outcomes, with analyses adjusted for potential confounders. The primary outcome will be fruit and vegetable (FV) intake (portions/day) as an indicator of diet quality. Data will then be used to model the impact of future policy change.

Phase I comprises recruitment of schools and students in order to collect a variety of quantitative data, including survey and dietary intake data from students, direct observations of the school eating environments, and questionnaires to key staff members and parents. The large variation in FSM uptake seen across schools also offers the opportunity for deeper exploration of barriers and facilitators to FSM uptake and school food systems more generally. Phase II will, therefore, comprise a case study with a small number of schools that will be selected from the schools recruited in phase I. A series of focus groups with students, and interviews with both staff/school stakeholders and parents will be conducted.

### Study setting

The sampling frame for the study will comprise secondary schools located within Northern Ireland (NI) and the Midlands and bordering regions in England, including a total of 20 local authorities, which differ in population density, ethnic diversity, FSM eligibility criteria and the school system. Both include areas of high deprivation.^35 36^

Routine data from the Department for Education has been used to identify state secondary schools. Children aged between 11 and 15 years will be included. A total of 32 schools will be recruited into the study (n=16 in NI and n=16) in the Midlands. The inclusion criteria for schools are determined based on the percentage of children eligible for FSM. A cut-point of 20% for pupil FSM eligibility has been determined based on the 50th eligibility percentile for all secondary schools across NI and the Midlands, that is, schools must have FSM eligibility rates above 20%. School selection will be stratified according to recruitment site and percentage FSM uptake of those eligible (in thirds), but within these strata, schools will be randomly selected. We anticipate a range of school-level FSM uptake based upon available data at the time of study planning, for example, in the Midlands, the mean FSM uptake was 86% (SD=20%, range 3%–100%; IQR 73%–99%) and in NI the mean FSM uptake was 77% (SD=12%; range 42%–100%; IQR 74%–85%).^37^,^39^

### Sample size calculation

A simplified power calculation was initially conducted dichotomising school-level FSM uptake into high and low. To detect a difference in FV portions/d of 0.5 between the high and low FSM uptake groups, assuming an SD of 1.9 (pooled SD from the Food provision, cUlture and Environment in secondary schooLs (FUEL) study,^40^ NI schools^41^ and the National Diet and Nutrition Survey^42^ with 90% power at 5% significance, we would require data from 720 students from 16 clusters (schools) in each group (cluster size n=45; total schools n=32; total n=1440). This was calculated using an intraclass correlation coefficient (ICC) of 0.03 (a conservative estimate based on data from the FUEL study and assuming balanced cluster size).^43 44^

However, in the primary analysis, we will investigate the linear increase in outcome per 10% point increase in school-level FSM uptake and hence will have greater power than suggested by the initial simplified calculation. We will also have 80% power to detect the following differences in diet quality (3.5%), free sugars (8 g) and fibre (1.1 g) intake, as secondary outcomes.

### Recruitment

Schools will be invited to take part in the study by sending an invitation letter to the school principal/headteacher, via post and email. This email will contain the school participation information sheet. Interested schools will contact the study team with a completed form or verbal communication that they are interested in participating in the study (expression of interest). A memorandum of understanding will be signed between the study team and the participating school. At this point, a school liaison person will be established both at the school and in the study team. The school liaison will be asked to either complete or nominate a member of staff to complete a Key School Information questionnaire. In this questionnaire, the respondent will provide key details about the schools and nominate staff members responsible for food provision to complete a role-specific questionnaire. At each participating school, one or two classes from each of two school years (years 7 and 10 Midlands, which are years 8 and 11 in NI) will be selected by the school for inclusion in the study. Once data collection is complete, each school shall receive a short report, containing a school-specific data summary, as well as £500 as a thank you for time spent participating in the study and an additional £5 for each parent questionnaire completed.

For phase 1 of the study, student data collection will be organised with the school liaison following a recruitment meeting to set up the required logistics. At least 7 days prior to the first student data collection, parents will receive a ‘parent information sheet’ which will contain an ‘opt-out of study form’. Schools will be asked to distribute the information sheets to parents via their usual communication routes, with flexibility for parent apps, email or hard copy. Students will also receive their own ‘student information sheet’ as a hard copy to keep. All students whose parents have not opted them out will be invited to take part in the study on the first data collection day, and to complete two data collection sessions conducted on separate days. Assent will be sought from each student, electronically, prior to completion of the first survey. Parents of students who participate in the study will then be invited to participate in a parent questionnaire, to be completed online or via hard copy. Students who complete the data collection will receive a £5 voucher, while each parent will receive a £15 voucher.

For phase II of the study (case study), qualitative work will be undertaken with already recruited schools (n=6–8), representative of FSM uptake levels; data collected during phase I will be used within the case study and will inform the sampling strategy to ensure representation of a wide range of schools within the case study sample.

Parents, staff and students will be asked to indicate interest in the phase II case study at the time of quantitative data collection and will then be selected and invited to participate; if the interest in participation is low, additional students, parents and staff will be recruited via the school through consultation with the school liaison. Selection will ensure a range of characteristics we will seek to include, for example, parents of those eligible and not eligible for FSM, those who seem to be experiencing food insecurity but who are not eligible for FSM; those whose children eat school lunch and packed lunch and those who are eligible for FSM but whose children do not eat school lunch. Information on these characteristics will have been collected during phase I. Students and parents who take part in phase II will again receive a £5 and £15 voucher, respectively.

School and student data collection commenced in October 2023; all schools were recruited within the 2023–2024 school year, with phase I data collection to complete by end of February 2025. Phase II case study selection and data collection commenced in December 2024 and will complete by the end of March 2025.

### Data collection methods

**Figure 1:**
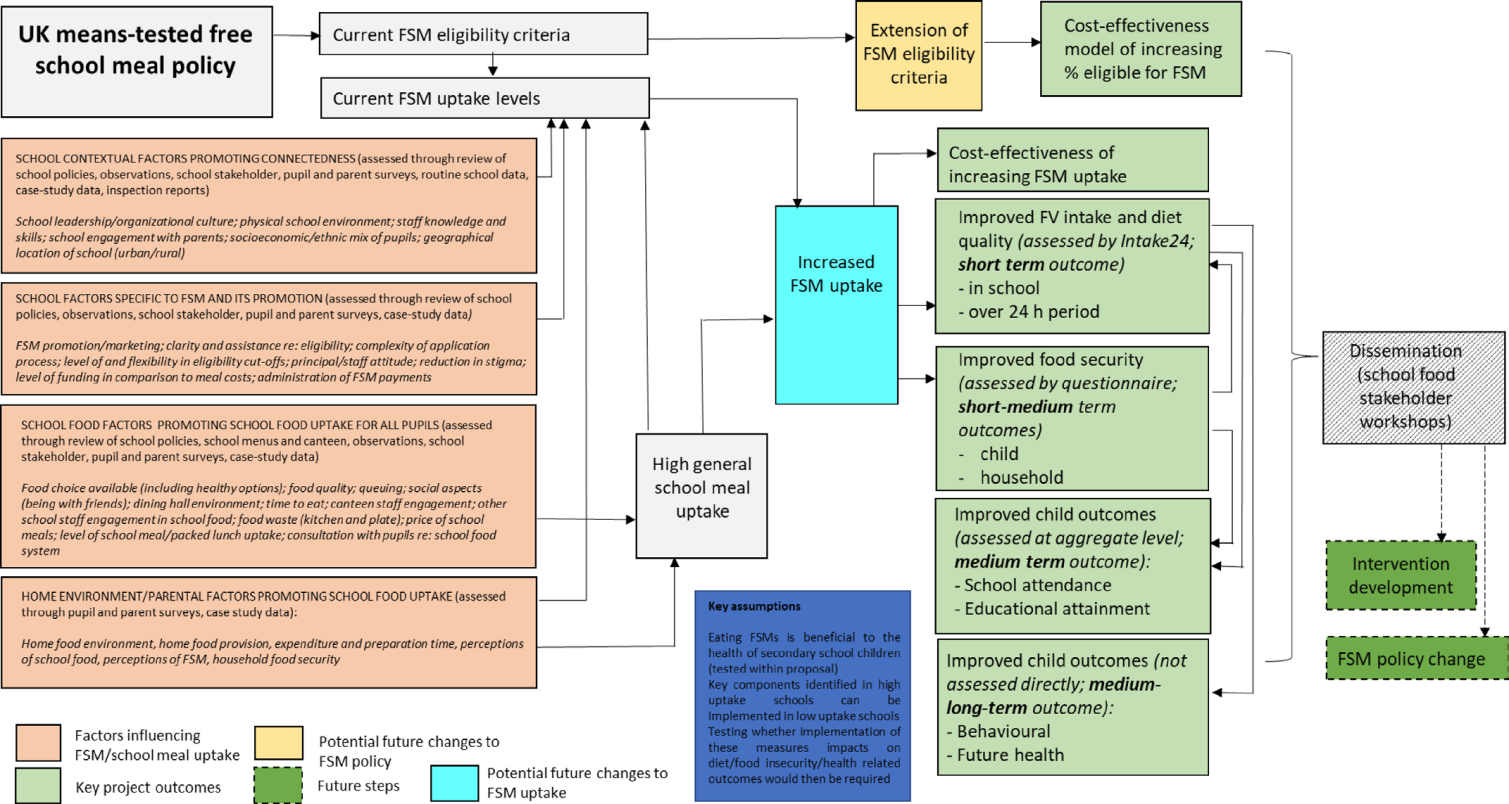
Logic model and theory of change describing the influence of the UK means-tested free school meal (FSM) policy on children’s dietary intake, diet quality and food insecurity outcomes, as well as factors influencing FSM uptake. FV, fruit and vegetable.

A logic model (figure 1) was developed, based on published literature describing the ways in which increased FSM uptake could lead to improved outcomes. Data collection methods were developed based on this logic model and for phase I include self-administered questionnaires with students, parents and stakeholders, school-level information including document review, individual-level and aggregated student information supplied by the schools, school food environment observations and interviews with school stakeholders (to collect quantitative data). For phase II, data collection methods are focus groups (students) and semistructured interviews (parents and school stakeholders). Data collection methods are detailed below and summarised in table 1.

**Table 1:** Data collection methods in phase I and phase II of the CANTEEN study

Phase I (quantitative)		
Data collection tool	Purpose	
Pupils		
Intake24	A 24-hour recall to capture pupil dietary intake A 24-hour recall to capture pupil dietary intake FV intake over 24 hours captured through an online 24-hour recall method; FV intake at school; Meeting 5 a day recommendations; intake of total energy, dietary fibre, free sugars and other key micro- and macronutrients at school and over 24 hours	Visit 1+Visit 2
Sociodemographic information	Name, school year group, age, ethnic background, gender, home postcode	Visit 1
Pupil online diet survey	Survey to capture FSM eligibility and uptake, usual school lunch consumption and money spent on food outside of school	
Child Health Utility 9D (Paediatric Quality of Life)	To capture pupil Quality of Life	
9 item Child Food Security Survey Module	To measure pupil food insecurity	
SIMBA Questionnaire	To measure pupil physical activity (1-item tool) SIMBA has been validated in the adolescent population. With a suggested modification to categorise minutes of activity in the past week as opposed to the number of days on which they achieved 30 min or more.	
Parents		
Parent Questionnaire	FSM eligibility, child’s usual food choices, FSM perceptions, usual home food practices and expenditure	
18-item household food security module	To capture FSM eligibility, child’s usual school food choices, school FSM system perceptions, usual home food practices and expenditure	
School		
Teacher Questionnaire; Senior Leadership Staff Questionnaire; School Governor Questionnaire; Catering Staff Questionnaire	Exploring FSM implementation, wider contextual/school food system influences (school leadership, school/parent engagement, pupil consultation mechanisms)	
Key School Information Survey	To capture information on the school food environment, food provision, food education, FSM data and collection of relevant school documents and policies	
Business Manager Questionnaire	To capture information relating to costs incurred by the school for food provision, eating environments, activities and facilities that the school has to support	
School Food Environment Observation Tool	To enable direct observation of school eating environment	
Key Pupil Information Form	To capture information on pupil’s educational attainment, whether they are eligible to receive FSM, whether English is an additional language for them, and if they are registered as having any special educational needs	
Observation and Report Evaluation Form	To capture feedback from schools (anonymously) to assist us in improving future efforts in our ongoing work with schools (non-compulsory component)	

**Phase II (qualitative)**	
**Data collection tool**	**Purpose**
Pupils	
Creative focus groups	To assess barriers and facilitators to FSM
Parents	
Semi-structured interview schedule	To assess barriers and facilitators to FSM
School	
Teacher semistructured interview schedule; senior leadership staff semistructured interview schedule; school governor semistructured interview schedule; catering staff semistructured interview schedule; business manager semistructured interview schedule	To assess barriers and facilitators to FSM and characterise schools with different levels of FSM uptake
Key Pupil Information Form*	To capture information on pupil’s educational attainment, whether they are eligible to receive FSM, whether English is an additional language for them, and if they are registered as having any special educational needs

* For new pupils, that is, those who do not take part in phase I.

FSM, free school meal; SIMBA, single-item minutes-based assessment.

### Phase I data collection

#### Student outcomes

Dietary intake will be collected at each of two student data collection visits, using an online self-completion 24-hour dietary recall tool called Intake24.^45^ This is a validated dietary assessment tool, having been assessed to give close estimates of both macro and micronutrient intakes, within the specific age groups being included in this study.^46^,^48^ Intake24 is the dietary assessment platform used by the UK National Diet and Nutrition Survey.^12^ Students will complete each 24-hour recall during timetabled sessions with researchers present on non-consecutive school days. From the Intake24 data, FV intake will be calculated (according to National Diet and Nutrition Survey methods^49^) over a 24-hour period at school as well as outside of school. Other dietary measures that will be explored as secondary outcomes will include calculation of diet quality^50^, meeting the 5-a-day FV recommendation, intake of energy, dietary fibre, free sugars and other key micronutrients and macronutrients. FV will be measured as portions (for FV) and nutrients as µg/mg/g and also expressed as a percentage of energy intake. Consumption will be explored at school and over 24 hours. Dietary intake variables will be averaged over the 2 days of data collection to account for daily variation.

Food insecurity will be a further secondary outcome and will be measured at the first data collection visit using the 9-item Child Food Security Survey Module, which has been validated in adolescents.^51 52^ At this visit, data will also be collected on demographics (age, name, DOB (for linkage to parent outcomes only), gender, ethnicity) and postcode (Index of Multiple Deprivation), usual school lunch consumption (ie, school food or food brought in from home or purchased outside of school), FSM eligibility and uptake, money spent on food outside of school, quality of life (Child Health Utility 9D (CHU-9D))^53^ and physical activity (Single-item minutes based assessment^54^). Students will be asked at this stage whether they are willing to be contacted for future focus group participation if their school is later selected as a case study school. The total data collection time for the first visit will be approximately 60–90 min, and for the second visit, approximately 30 min.

Key student information regarding attainment, attendance, first language, Special Educational Needs (SEN) registration status and FSM status will be requested from each school for all participating students.

#### Parent outcomes

Parents/guardians of participating students will be contacted to complete a parent questionnaire. This will include an 18-item household food security measure,^55^ FSM eligibility and their children’s usual food choices at school. These data will be cross-linked to similar data collected from students, therefore, allowing for triangulation of responses where required. The questionnaire will also include questions on school FSM system perceptions, usual home food practices and food expenditure. Data on FV intake and knowledge will be collected as an indicator of nutritional knowledge.

#### School outcomes

School level data capture will include a direct observation of the school eating environment, including areas where food is served at break and lunchtime. Modified observation checklists that were originally developed by the FUEL study^56^ based on the School Food Plan^57^,^59^ will be used to collect these data.

Staff questionnaires will be distributed to members of staff including the school business manager, catering manager, governors, teachers and senior leadership who play a key role within food provision, food education and FSM administration. These will be administered using an online platform (or paper format if preferred) to explore (1) FSM implementation and (2) wider contextual/school food system influences (school leadership, school/parent engagement, student consultation mechanisms). Support to complete the questionnaires will be provided where required by the research team. The catering manager and business manager will be invited to self-complete the first part of their respective questionnaire and then invited to a 1-hour, online or face-to-face, interview to confirm responses provided in part one and complete a more detailed second part to allow the comprehensive collection of cost data.

Additionally, the school will also be asked to provide key documents including policies and routinely collected data, including FSM promotion, eligibility, registration processes, school food and other relevant policies, school meal data (including uptake), aggregated attendance and educational outcome data (General Certificate of Secondary Education (GCSE)) and relevant inspection reports.

### Phase I data analysis

Focusing on the 32 study schools, multilevel models will be developed to estimate the linear increase in student outcomes per 10% point increase in FSM uptake at school levels, accounting for clustering (schools) and adjusted for both the routinely observed school-level and student-level characteristics (including FSM eligibility). Given these adjustments, the increase in student outcomes per 10% point increase in FSM uptake will reflect FSM uptake that is potentially modifiable by school-level policy. A further exploratory analysis will consider FSM uptake and association with aggregated (at school level) attendance and educational outcomes.

In addition to the main analyses, which will include all students, regardless of FSM eligibility, multilevel linear regression models will be used to calculate the mean difference in outcomes (including diet and food insecurity) comparing students taking FSM and those not taking FSM within FSM-eligible participants only, adjusting for school-level and student-level characteristics and clustering.

### Economic evaluation

We will undertake a cost–utility analysis of increasing FSM participation. This will be exploratory due to the expected variation in costs and models of FSM implementation, and ranges in assumptions about the persistence of changes in diet and impacts on future health. The resulting estimates will be used to evaluate the cost-effectiveness of FSM participation at increasing quality-adjusted life-years (QALYs). Costs will be calculated as the marginal costs of increasing FSM participation, minus future National Health Service (NHS costs that are foregone as a result of forecast changes in future health. These will be expressed in 2024 pounds sterling. Utility gains will be calculated as the sum of contemporaneous changes in QALYs derived from student completion of the CHU-9D during primary data collection, plus future gains in QALYs estimated based on forecast changes in future health.

### Phase II data collection

Interviews will be undertaken with school stakeholders (eg, catering and business managers; governors; teachers, senior leaders and other relevant school staff; n=4/school) and parents (n=4–8/school), using semistructured topic guides. The aim of these will be to explore views of the FSM system, school and home food environment, and broader school contextual factors (eg, capacity constraints and additional investment requirement to enable higher levels of take-up). Interviews will be offered either via telephone, online or in person, as per the preference of parents and school staff. Each interview will take around 1 hour. Parents will be asked to complete a short survey including questions on ethnicity, gender, age group, child FSM eligibility and family food insecurity after they have signed the consent form if they have not already participated in phase I of the study.

Focus groups will be conducted with students (n=2–4 focus groups/school; n=6–8 students/group invited to ensure participation of n=4–5), ensuring representation of participants both eligible and ineligible for FSM. Scheduling of focus groups will try to ensure discussion among pupils with similar characteristics, for example, age and FSM status. In the focus groups, we will employ creative methods, using characters from the Disney Pixar film, Inside Out 2, sourced from the Be Happy resources,^60^ with the aim of exploring in depth the emotions associated with school food and the school eating environment. Questions will be incorporated within the exercise using hypothetical scenarios to allow consideration of a range of student perspectives, for example, students who take and do not take school meals and who are eligible or not eligible for FSM. Students will be recruited from the classes in which data collection occurred for the quantitative study, with expansion as required should recruitment targets not be met. Focus groups will take place in the school on an agreed date and time. Each focus group will take approximately 1.5 hours.

The school will be asked to provide FSM eligibility and SEN status, gender, age and ethnicity for students who complete the case study and who have not already participated in phase I. Prior to the focus group discussions, all students will be asked for their assent. After providing assent, they will also be asked to state their year group and answer a question about their usual lunchtime eating routine (eg, packed lunch/school lunch/mixed). Interviews and focus groups will be audio-recorded.

### Phase II data analysis

Interviews will be transcribed, anonymised and checked for accuracy by the research team; focus groups will be similar except they will be transcribed by an external transcription service. Thematic analysis techniques will be employed,^61^ which seek to identify and classify the content of qualitative data, to explore patterns and differences across interviews and focus groups, with the aim of providing explanatory conclusions clustered around themes. The transcripts will be coded, then collated into themes and subthemes according to the conceptual similarity of codes. Agreement on concepts and coding will be sought between members of the research team (including across recruitment sites) throughout the analysis process to ensure reliability. A proportion of the data (20%) will be coded by two different team members to check for inter-coder reliability. Thematic analysis will be supported by qualitative analysis software (NVivo).

The sequential nature of the quantitative and qualitative data collection will potentially allow a mixed-methods approach drawing on sequential explanatory design.^62^,^64^ In this way, the qualitative data collection can help explain, or elaborate on, the quantitative results obtained. The quantitative data and their subsequent analysis will provide a general understanding, with the qualitative data and their analysis refining and explaining the results by exploring participants’ views in more depth.^62^,^64^ School factors across the different levels of uptake will be explored, as well as commonalities and differences in factors related to FSM provision and support, allowing characterisation of schools according to these different levels of uptake.

### Patient and public involvement and engagement

Patient and public involvement and engagement (PPIE) was integral to the initial research question development; school staff reported concern about children’s nutrition and the effectiveness of the current FSM policy, particularly in light of COVID-19 and the changes being seen in those eligible for FSM. Similar concerns were expressed in surveys of school food system stakeholders undertaken as part of the GENIUS school food network.^65^ Secondary school students, parents and school staff and principals were advised on recruitment and data collection methods and levels of monetary rewards in order to make the project appealing to schools, parents and students. They also gave feedback on the school report format which is being used to encourage school participation and will provide schools with specific information on the current school food system including feedback on student and parental views of school food. A secondary school principal (SR; PPI Co-I and on Study Management Group (SMG)) advised on various approaches to engage schools and students, encouraging parental completion of questionnaires and accessing school environment/management data.

A separate PPIE subgroup including parent, school staff and student group representation has been recruited and has met regularly during the planning stages (discussing recruitment and outcome data collection) and during the different data collection phases. They have also assisted with pilot testing of all outcome measures. Their views are fed into the Study Steering Committee (SSC) and SMG by PPIE representatives on both, and there is reciprocity, with both SSC and SMG discussions being fed back to the PPIE subgroup. The PPIE representatives will help shape the dissemination plan and develop the dissemination materials to ensure effective communication of findings to users and stakeholders. Thus, PPIE is occurring at all project stages and adhering to national standards.^66^ PPIE group members have received training and all input is supported accordingly, with reimbursement in the form of shopping vouchers.

### Data collection tool piloting

The student, school staff, governor, student and parent questionnaires were all piloted with members of the PPIE panel in the relevant groups. Similarly, case study interviews and focus group schedules have all been piloted through the same process. The tools were refined based on the feedback provided.

This research will provide critical evidence on the impact of a key UK policy that is designed to mitigate inequalities by evaluating the current, means-tested FSM policy on diet quality and food insecurity outcomes in secondary school students in the UK. The study design includes both quantitative and qualitative research to provide a thorough and in-depth analysis understanding of the effectiveness of FSM policy. Economic evaluation will determine the cost-effectiveness of the current means-tested FSM policy. The study design includes both quantitative and qualitative research and will gather data from a wide range of stakeholders to explore the factors associated with FSM uptake and to provide a thorough and in-depth analysis and understanding of the effectiveness of FSM policy.

## Ethics and dissemination

Full ethical approval was obtained from Queen’s University Belfast Faculty of Medicine, Health and Life Sciences Research Ethics Committee on 3 July 2023 (MHLS 23_55). Ethical approval was affirmed by the University of Birmingham Ethical Review Committee (ERN_22-1447). The study is registered on the ISRCTN registry (ISRCTN 14009382; https://www.isrctn.com/ISRCTN14009382). For students, parents were contacted directly by the school and could opt their child out of participation, followed by student assent; informed consent was obtained from all other participants.

A range of outputs is anticipated: peer-reviewed publications, conference presentations for academic audiences, policy briefings for government, public health bodies and for those responsible for school food policy development, guidance and summary report/materials for schools (and to be shared with their stakeholders, eg, students, parents) and research summaries for non-academic audiences including the media. In particular, we will focus on the potential suggested options for changes to the FSM policy and what our data suggest are the implications of those changes as well as the modifiable factors associated with FSM uptake. Our final objective is to share findings via stakeholder workshops, with the purpose of refining the logic model, identifying key aspects of successful FSM uptake and guiding future school policy and interventions. Further dissemination plans will be guided by PPIE. After publication of the main study findings, anonymised data will be available on request from the study Chief Investigator.

### Data management and study oversight

Queen’s University Belfast (QUB) is the study sponsor and data controller for this study and assumes overall responsibility for the study. Data management and storage is compliant with the UK Data Protection Act 2023 and adheres to QUB’s policies and procedures. At the end of the study, each site will send original source documentation to QUB for archive. Study data will be anonymised following data matching (eg, students and parents) and stored securely for 10 years.

An independently chaired SSC has been convened to provide study oversight. Membership comprises three independent academics with relevant expertise, a representative from public health, a representative from regional local government with responsibility for school food, a public representative, the chief investigator and a further Site Lead. The committee approved the study protocol and has had sight of and the opportunity to discuss proposed amendments.

### Strengths and limitations of this study

This research will provide critical evidence on the impact of a key UK policy that is designed to mitigate inequalities by evaluating the current, means-tested FSM policy on diet quality and food insecurity outcomes in secondary school students in the UK. The study design includes both quantitative and qualitative research to provide a thorough and in-depth analysis understanding of the effectiveness of FSM policy. Economic evaluation will determine the cost-effectiveness of the current means-tested FSM policy. The study design includes both quantitative and qualitative research and will gather data from a wide range of stakeholders to explore the factors associated with FSM uptake and to provide a thorough and in-depth analysis and understanding of the effectiveness of FSM policy.

However, there are a number of limitations to consider. The current nature of the policy precludes the use of randomised controlled trial methodology, and therefore, the design is observational and cross-sectional in nature, which limits inferences about the causality in the relationship between FSM participation, diet quality and food security. The study is based on self-report questionnaires for students, parents and staff members and, particularly for the dietary data from students, is susceptible to reporting and recall bias leading to a decrease in data validity. Fieldwork will only be conducted in two geographical areas of the UK (Midlands and NI), although these are diverse, and so findings may not be generalisable to the wider context of the UK as there may be socioeconomic/cultural differences which affect the uptake of FSM. Canteen observations, although collected using standardised templates and methodology, could potentially be biased by researchers’ interpretations. The limited coverage of the case study schools (n=6–8) may not provide the full spread of contextual factors that influence FSM uptake. Finally, the economic assessment, although informative, may not capture nuanced social and cultural factors driving the consumption of diet and food insecurity.
