# Supplementary material for: Free school meals, diet quality and food insecurity in secondary school students: protocol for a multiple-methods study – the CANTEEN study
Source: BMJ Open. 2025 Oct 20;15(10):e101428. doi: 10.1136/bmjopen-2025-101428 (PMC12542528; doi:10.1136/bmjopen-2025-101428)
Supplement: Supplementary file 1 [file bmjopen-2025-101428.reviewer_comments.pdf]

PEER REVIEW HISTORY

BMJ Open publishes all reviews undertaken for accepted manuscripts. Reviewers are asked to complete a checklist review form and are provided with free text boxes to elaborate on their assessment. These free text comments are reproduced below.

ARTICLE DETAILS

Title (Provisional)

The CANTEEN Study: free school meals, diet quality and food insecurity in secondary school students: protocol for a multiple-methods study

Authors

Alving-Jessep, Emma; Pallan, Miranda; Ansell, Ellie; Hamill, Lesley; McConnell, Cara; McIlwee, Desiree; McKinley, Michelle C.; Moore, Sarah E.; Murphy, Marie; Neville, Charlotte; O'Neill, Christina; Sevel, Estera; Adab, Peymane; Bryant, Maria; Chambers, Stephanie; Cardwell, Christopher R; Ensaff, Hannah; Evans, Charlotte; Reid, Stephen; Holford, Angus; Bradley, Jennifer; Spence, Suzanne; Woodside, Jayne

---

VERSION 1 - REVIEW

**Reviewer**	**1**
**Name**	**Altemimi, Ammar**
**Affiliation**	**University of Basrah College of Agriculture**
**Date**	**02-Sep-2025**
**COI**	**None**

In an important study such as this, there are however several limitations to consider. First, the study is based on self-reported questionnaires for students, parents and staff members and is susceptible to reporting and recall bias leading to a decrease in data validity. This is only conducted on 2 areas (and Midlands and Northern Ireland), and so the wider context of the UK would not be able to be generalised as there may be socio-economic/ cultural differences which affect the take up of Free School Meals (FSM). Furthermore, observations of schoolyard environments could potentially have been biased by researchers' interpretations. The limited coverage of the case study schools (n=6–8) may not provide the full spread of contextual factors that influence FSM take-up. In addition, the cross-sectional design of the primary data collection limits inferences about the causality in the relationship between FSM participation, dietary quality and food security. Lastly, the economic assessment, although informative, will not capture nuanced social and cultural factors driving the consumption of diet and food insecurity.

VERSION 1 - AUTHOR RESPONSE

Reviewer: 1
Dr. Ammar Altemimi, University of Basrah College of Agriculture

Comments to the Author:
In an important study such as this, there are however several limitations to consider. First, the study is based on self-reported questionnaires for students, parents and staff members and is susceptible to reporting and recall bias leading to a decrease in data validity. This is only conducted on 2 areas (and Midlands and Northern Ireland), and so the wider context of the UK would not be able to be generalised as there may be socio-economic/ cultural differences which affect the take up of Free School Meals (FSM). Furthermore, observations of schoolyard environments could potentially have been biased by researchers' interpretations. The limited coverage of the case study schools (n=6–8) may not provide the full spread of contextual factors that influence FSM take-up. In addition, the cross-sectional design of the primary data collection limits inferences about the causality in the relationship between FSM participation, dietary quality and food security. Lastly, the economic assessment, although informative, will not capture nuanced social and cultural factors driving the consumption of diet and food insecurity.

A section has been included, as per the Editor and reviewer's request.

Reviewer: 1
Competing interests: Not applicable
